# Elderly Patients With Proximal Femoral Fractures and SARS-CoV-2 Infection: An Observational Study on 30-Day Postoperative Mortality

**DOI:** 10.7759/cureus.94861

**Published:** 2025-10-18

**Authors:** Luigi Meccariello, Giuseppina Casolaro, Vincenzo Boniello, Giovanna Di Salvio, Pascasio Di Blasio, Andrea Balzano, Pietro Crocco, Melania D'Agostino, Federica Innaurato, Luigi Matera

**Affiliations:** 1 Department of Orthopedics and Traumatology, Azienda Ospedaliera (AO) San Pio, Benevento, ITA; 2 Financial and Management Department, Azienda Sanitaria Locale (ASL) Benevento, Benevento, ITA; 3 Neuroreanimation Unit, Department of Neuroscience, Azienda Ospedaliera (AO) San Pio, Benevento, ITA; 4 Anesthesiology and Reanimation Unit, Azienda Ospedaliera (AO) San Pio, Benevento, ITA

**Keywords:** covid-19, geriatric, hip, infection, mortality, outcomes

## Abstract

Purpose: Hip fractures in older adults require multidisciplinary care and are associated with high morbidity and mortality. The COVID-19 pandemic has particularly impacted this vulnerable population. This study evaluated the 30-day outcomes of elderly patients with hip fractures and SARS-CoV-2 infection treated in a single center.

Methods: We conducted a retrospective cohort study including patients admitted between July 2020 and December 2022 with proximal femoral fractures. Demographic data, comorbidities, COVID-19 status, and postoperative outcomes were collected from electronic records. Patients were categorized into four groups according to COVID-19 status and surgical timing (≤48 h vs. >48 h). Mortality, complications, and hospital stay were analyzed.

Results: A total of 185 patients (mean age 80 years) were included; 56 (30.3%) tested positive for COVID-19. COVID-19-positive patients had significantly higher comorbidity scores and required postoperative oxygen therapy more frequently. Thirty-day mortality was markedly higher among infected patients (OR 7.01; 95% CI 4.94-8.77; p<0.00001). They also showed increased in-hospital mortality, complications, and prolonged hospitalization.

Conclusion: SARS-CoV-2 infection significantly worsened short-term outcomes in elderly patients with hip fractures. A coordinated multidisciplinary approach remains crucial to reduce mortality and improve survival in this fragile population.

## Introduction

During the initial years of the SARS-CoV-2 (COVID-19) pandemic, no standardized anesthetic protocol was established for elderly patients undergoing surgery for hip fractures. The state of emergency declared in 2020 profoundly altered hospital practices, prompting investigations into potential differences in anesthetic management and survival outcomes for patients undergoing hip fracture repair with gamma nail osteosynthesis, compared with the pre-pandemic year 2019 [1].

The global COVID-19 pandemic was associated with excess mortality, particularly among older adults with multiple comorbidities. Patients with proximal femoral fractures are already known to have a high risk of mortality, reaching up to 30% within the first postoperative year [2].

In a large cohort study, Kamalapathy et al. [3] analyzed 1,280 patients with a confirmed COVID-19 diagnosis matched against 3,831 non-COVID-19 patients. Those with COVID-19 demonstrated an increased risk of postoperative complications, including pneumonia, acute kidney injury, urinary tract infection, and readmission following total joint arthroplasty (TJA), compared with their non-infected counterparts. However, no significant differences in complication rates were observed between vaccinated and unvaccinated COVID-19-positive patients undergoing TJA.

Overall, the COVID-19 pandemic has significantly impacted outcomes in this high-risk population. The present study aims to describe the clinical outcomes of hip fracture patients with concomitant COVID-19 infection treated at a single tertiary care center.

## Materials and methods

This retrospective cohort study included patients admitted with proximal femoral fractures between July 2020 and December 2022. It was conducted at Azienda Ospedaliera (AO) San Pio Hospital, Benevento, Italy, after obtaining approval from the Internal Committee of Azienda Ospedaliera (AO) San Pio (approval number: #2023-0012-studio-retrospettivo). Data were obtained from electronic medical records, including patient demographics, comorbidities, COVID-19 status, and short-term clinical outcomes.

Inclusion and exclusion criteria

Inclusion criteria were as follows: age ≥65 years (both male and female), deferred emergency or scheduled surgery, proximal femoral fractures, and surgical treatment with short or long gamma nails, plate and screws, total hip arthroplasty, or hemiarthroplasty. In contrast, exclusion criteria included the following: psychiatric or neoplastic diseases, conservative (non-surgical) fracture management, age <65 years, and multiple trauma.

A total of 185 patients met the eligibility criteria. The cohort was stratified into four groups based on COVID-19 status and surgical timing: ≤H48 SARS-CoV-2 Negative, non-COVID-19 patients operated within 48 hours of trauma; >H48 SARS-CoV-2 Negative, non-COVID-19 patients operated later than 48 hours after trauma; ≤H48 SARS-CoV-2 Positive, COVID-19-positive patients operated within 48 hours of trauma; and >H48 SARS-CoV-2 Positive, COVID-19-positive patients operated later than 48 hours after trauma.

We set time zero for the evaluation of outcomes, hospitalization, and the duration of surgery and complications from the moment the patient was admitted to the emergency room. 

Outcomes

The primary outcomes included in-hospital mortality, time from surgery to death (in days), mortality within 30 days post-discharge, American Society of Anesthesiologists (ASA) score of deceased patients, and Diagnosis-Related Group (DRG) costs (in euros). Secondary outcomes included complication rate and length of hospital stay.

Statistical analysis

Data were processed using Microsoft Excel (Microsoft Corporation, Redmond, Washington, United States) and analyzed using standard statistical methods. Descriptive statistics (mean±standard deviation) were used to summarize continuous variables. Continuous variables were compared using Student's t-test. Categorical variables were compared using the chi-squared test or Fisher's exact test (for subgroups with <10 patients). Statistical significance was set at p<0.05. The Pearson correlation coefficient (r) was calculated to assess associations between predictive scores and outcomes. Age was rounded to the nearest year; outcome predictive scores were approximated to the first decimal, and correlation coefficients to the second decimal. Multivariate regression analysis was performed to compare the COVID-19 cohort with controls, adjusting for relevant covariates.

## Results

A total of 185 patients were included in the study, with a mean age of 80 years. Of these, 56 patients (30.3%) tested positive for COVID-19 infection in the preoperative period. Patients were divided into four groups (Table 1): ≤H48 SARS-CoV-2 Negative, non-COVID-19 patients operated within 48 hours of trauma (n=90); >H48 SARS-CoV-2 Negative, non-COVID-19 patients operated later than 48 hours (n=39); ≤H48 SARS-CoV-2 Positive, COVID-19-positive patients operated within 48 hours (n=30); and >H48 SARS-CoV-2 Positive, COVID-19-positive patients operated later than 48 hours (n=26).

**Table 1:** Clinical details of 185 proximal femoral fractures

Groups	≤H48 SARS-CoV-2 Negative	>H48 SARS-CoV-2 Negative	≤H48 SARS-CoV-2 Positive	>H48 SARS-CoV-2 Positive	P-value
Number of patients	90	39	30	26	-
Average age of patients (SD)	81.8±11.0	81.6±11.3	79.6±11.7	78.8±12.6	>0.05
Range of age of patients	(65.4-101.6)	(65.4-102.6)	(65.6-95.7)	(65.2-93.4)	>0.05
Gender ratio (M:F)	0.58 (33:57)	0.54 (14:25)	0.58 (11:19)	0.56 (14:25)	>0.05
Type of accident: number (%)	Domestic fall: 70 (77.78%)	Domestic fall: 30 (76.93%)	Domestic fall: 21 (70%)	Domestic fall: 18 (69.23%)	>0.05
	Traffic accident: 10 (11.12%)	Traffic accident: 3 (7.69%)	Traffic accident: 4 (13.33%)	Traffic accident: 3 (11.54%)	>0.05
	Work accident: 5 (5.55%)	Work accident: 3 (7.69%)	Work accident: 3 (10%)	Work accident: 3 (11.54%)	>0.05
	Other accident: 5 (5.55%)	Other accident: 3 (7.69%)	Other accident: 2 (6.67%)	Other accident: 2 (7.69%)	>0.05
Type of femoral fractures: number (%)	Femoral neck: 36 (40%)	Femoral neck: 16 (41.02%)	Femoral neck: 12 (40%)	Femoral neck: 10 (38.46%)	>0.05
	Pertrochanteric: 36 (40%)	Pertrochanteric: 16 (41.02%)	Pertrochanteric: 12 (40%)	Pertrochanteric: 10 (38.46%)	>0.05
	Subtrochanteric: 18 (20%)	Subtrochanteric: 7 (17.96%)	Subtrochanteric: 6 (20%)	Subtrochanteric: 6 (23.08%)	>0.05

There were no statistically significant differences between the groups with respect to demographic variables such as mean age and sex distribution (Table 1). The H48 SARS-CoV-2 Negative group showed a slightly higher mean age compared to the other groups. Domestic falls represented the most frequent cause of trauma across all groups, followed by traffic accidents. The most common fracture types were femoral neck and lateral femoral neck fractures, followed by subtrochanteric fractures (Table 1).

A statistically significant difference was observed in mean waiting time for surgery (p<0.001; Table 2). Patients in the ≤H48 SARS-CoV-2 Negative group underwent surgery after a mean of 1.8±0.2 days, compared with 4.5±0.2 days in the >H48 SARS-CoV-2 Negative group, 1.9±0.1 days in the ≤H48 SARS-CoV-2 Positive group, and 7.0±5.4 days in the >H48 SARS-CoV-2 Positive group.

**Table 2:** Clinical results for each of the four groups The asterisk (*) indicates the group that had a statistically significant difference from the others. ASA: American Society of Anesthesiologists

Groups	≤H48 SARS-CoV-2 Negative	>H48 SARS-CoV-2 Negative	≤H48 SARS-CoV-2 Positive	>H48 SARS-CoV-2 Positive	P-value
Average days for surgery (SD, range)	1.8±0.2 (1-2)*	4.5±0.2 (3-12)	1.91.8±0.1 (1-2)	7±5.4 (6-24)	*<0.001
Mean length of stay in days in hospital (SD, range)	6.7±3.2 (2-21)*	10.7±6.5 (3-25)	26.6±13.1 (3-45)	29.7±13.1 (3-54)	*0.046
Total number of deceased patients during stay in hospital (%)	2 (2.22%)	5 (12.82%)	12 (40%)*	8 (30.77%)	*<0.001
Mean postoperative period to death in days (SD, range)	3.5±2.1 (2-5)*	6.5±3.4 (2-11)	16.75±13.71 (2-43)	10±7.21 (2-23)	*<0.001
Total number of deceased patients after 30 days from the hospital discharge (%)	20 (22.22%)	5 (12.82%)*	12 (40%)	8 (30.77%)	*<0.001
Total number of deceased patients (%)	22 (24.44%)	10 (25.64%)	24 (61.53%)*	16 (61.54%)*	*<0.001
Median ASA score of deceased patients (range)	3 (2-4)	3 (2-4)	3 (2-4)	3 (2-5)	0.779
Median hospitalization cost of patients in euros (SD, range)	7004 (±862; 5438-7953)	6823 (±1055; 5438-7953)	*15423	*15423	*0.024

Length of hospital stay also differed significantly between groups (p=0.046; Table 2). The ≤H48 SARS-CoV-2 Negative group had the shortest mean hospital stay (6.7±3.2 days), while the longest stays were recorded in COVID-19-positive patients: 26.6±13.1 days in the ≤H48 SARS-CoV-2 Positive group and 29.7±13.1 days in the >H48 SARS-CoV-2 Positive group.

In-hospital mortality was highest in the ≤H48 SARS-CoV-2 Positive group (40.0%), followed by the >H48 SARS-CoV-2 Positive group (30.8%), compared with 12.8% in the >H48 SARS-CoV-2 Negative group and 2.2% in the ≤H48 SARS-CoV-2 Negative group (p<0.001; Table 2).

The mean postoperative interval to death also showed significant differences (p<0.001; Table 2). The shortest interval was observed in the ≤H48 SARS-CoV-2 Negative group (3.5±2.1 days), whereas the longest was in the ≤H48 SARS-CoV-2 Positive group (16.8±13.7 days).

Thirty-day post-discharge mortality was significantly higher in COVID-19-positive groups (40% in ≤H48 SARS-CoV-2 Positive and 30.8% in >H48 SARS-CoV-2 Positive) compared with non-infected groups (22.2% in ≤H48 SARS-CoV-2 Negative and 12.8% in >H48 SARS-CoV-2 Negative; p<0.001; Table 2).

Total mortality was markedly higher in COVID-19 patients: 61.5% in both ≤H48 SARS-CoV-2 Positive and >H48 SARS-CoV-2 Positive groups, compared with 25.6% in the >H48 SARS-CoV-2 Negative group and 24.4% in the ≤H48 SARS-CoV-2 Negative group (p<0.001; Table 2). The median ASA score among deceased patients was 3 across all groups (p=0.779).

Hospitalization costs (DRG) were significantly higher for COVID-19-positive patients (mean €15,423) compared with €7,004 in the ≤H48 SARS-CoV-2 Negative group and €6,823 in the >H48 SARS-CoV-2 Negative group (p=0.024; Table 2).

Analysis of causes of death (Table 3) showed no significant group differences in rates of cardiac decompensation, pulmonary embolism, non-COVID-19 pneumonia, myocardial infarction, sepsis, gastrointestinal bleeding, epileptic shock with aspiration, or hepatic failure. Only COVID-19 pneumonia was significantly associated with mortality (p=0.023), occurring in 40% of the ≤H48 SARS-CoV-2 Positive group and 30.8% of the >H48 SARS-CoV-2 Positive group, while absent in non-infected patients.

**Table 3:** Common causes of post-discharge death

Groups	≤H48 SARS-CoV-2 Negative	>H48 SARS-CoV-2 Negative	≤H48 SARS-CoV-2 Positive	>H48 SARS-CoV-2 Positive	P-value
Cardiac decompensation with myocardial failure, n (%)	7 (7.78%)	2 (5.12%)	4 (13.33%)	1 (3.85%)	0.46
Pulmonary embolism, n (%)	2 (2.22%)	22 (5.12%)	1 (3.33%)	1 (3.85%)	0.77
Non-COVID-19 pneumonia, n (%)	4 (4.44%)	1 (2.56%)	1 (3.33%)	2 (7.7%)	0.36
COVID-19 pneumonia, n (%)	0	0	12 (40%)	8 (30.77%)	0.023
Acute myocardial infarction, n (%)	5 (5.56%)	1 (2.56%)	2 (6.67%)	1 (3.85%)	0.32
Sepsis, n (%)	1 (1.11%)	1 (2.56%)	1 (3.33%)	1 (3.85%)	0.77
Gastrointestinal bleeding, n (%)	1 (1.11%)	1 (2.56%)	1 (3.33%)	1 (3.85%)	0.77
Epileptic shock with aspiration, n (%)	1 (1.11%)	1 (2.56%)	1 (3.33%)	1 (3.85%)	0.77
Hepatic failure, n (%)	1 (1.11%)	1 (2.56%)	1 (3.33%)	0	0.77

COVID-19-positive patients had significantly higher Charlson comorbidity scores compared with non-infected patients (p=0.037 and p=0.045). A total of 31 COVID-19-positive patients required supplemental oxygen postoperatively, although no patients required mechanical ventilation or organ support.

Overall, COVID-19-positive patients had a significantly higher 30-day mortality risk (OR 7.01; 95% CI 4.94-8.77; p<0.00001). Secondary outcomes also revealed significantly higher inpatient mortality (OR 19.37; 95% CI 8.22-48.87), complication rate (OR 9.88; 95% CI 4.58-21.21), and length of hospital stay (mean difference 5.14 days; 95% CI 2.68-7.66) compared with non-infected patients.

## Discussion

The COVID-19 pandemic has led to significant mortality, severe health consequences, and unprecedented challenges in hospital care worldwide. In 2020, a reduction in surgical procedures for proximal femoral fractures was reported, largely due to restrictions on daily activities and measures implemented to contain viral spread [4-7]. For the same reasons, a lower average patient age and a higher incidence of domestic accidents were observed. The pandemic also created substantial barriers to medical care for elderly patients with hip fractures, resulting in delayed surgical access and higher complication rates [5,6]. Recent studies and meta-analyses consistently confirm that COVID-19 infection increases mortality risk in patients with hip fractures [1-7].

COVID-19 has been associated with higher risks of therapeutic failure compared to pre-pandemic scenarios. These risks are difficult to prevent and, in many cases, unavoidable, leading to exponentially increased hospital mortality rates. This trend has been partly attributed to advanced age and comorbidity profiles of the affected population [1-7]. Notably, many patients were admitted to "non-COVID" wards, facilities not specifically designed for SARS-CoV-2-positive patients, which may have further contributed to poor outcomes [5].

Zajonz et al. [8], in a study conducted before the introduction of SARS-CoV-2 vaccination, reported that over 40% of patients with proximal femoral fractures and concomitant infection died during hospitalization. Mortality was strongly influenced by patient age and by repeated positive SARS-CoV-2 tests. Furthermore, prolonged hospital stays in infected patients were linked to the limited availability of transfers to rehabilitation or skilled nursing facilities. Elderly, frail patients, therefore, represented a particularly vulnerable subgroup when hip fractures coincided with COVID-19 infection.

The risk of COVID-19-related mortality is known to increase with age [1-13]. Our findings align with previous reports from Italy [4-7,9], England [10], Spain [11], and Brazil [12], which all documented increased death rates during the pandemic. Elevated mortality and complication rates during hospitalization were consistently associated with COVID-19 infection [12-15]. In our cohort, the most frequent causes of death were hydroelectrolytic disorders and sepsis. Comparable studies similarly reported sepsis and electrolyte imbalance as major contributors to unfavorable outcomes among hip fracture patients treated during the pandemic [1-14]. A likely explanation is the reorganization of care pathways and increased workload placed on healthcare teams, which may have delayed treatment and compromised patient outcomes [15].

Meta-analyses further corroborate these findings. Wang et al. [16] reported a 32% mortality rate, with a relative risk of 5.66 (95% CI: 4.01-7.98; p<0.001) for postoperative mortality in SARS-CoV-2-positive patients compared to negative controls. More recently, Koschmeder et al. [17] suggested in a pilot cohort study that COVID-19 infection within 50 days of orthopedic surgery did not significantly increase the risk of complications such as deep vein thrombosis (DVT), pulmonary embolism, surgical site infection, renal failure, ICU admission, reoperation, or death, indicating that the timing and severity of infection may be critical factors.

In contrast, Tanaka et al. [18] found that, among non-COVID-19 patients, waiting time for surgery, hospital stay duration, and in-hospital complication or mortality rates did not significantly differ before and after the pandemic, although infection rates were higher during the early pandemic period. Similarly, Santander et al. [1] observed that the median length of hospital admission decreased significantly from 7.0 days in 2019 to 4.5 days in 2020. This reduction likely minimized the risk of nosocomial SARS-CoV-2 infection without increasing postoperative complications, facilitating earlier rehabilitation and reducing healthcare costs.

Importantly, delayed surgical intervention has been consistently recognized as an independent risk factor for increased postoperative mortality [19]. Thus, timely surgical management remains a cornerstone in the treatment of proximal femoral fractures, even in the context of a pandemic.

This study has some limitations that should be taken into account when interpreting the results. First, it is a single-center retrospective study, which may reduce the generalizability of the findings to other healthcare settings and more heterogeneous populations. Although the overall sample size (185 patients) is not negligible, it remains limited, particularly within subgroups (e.g., COVID-19-positive patients operated within 48 hours), thus lowering the statistical power of some analyses.

Second, data collection was based on electronic medical records and pre-existing clinical documentation, with the potential risk of incomplete or inaccurate reporting. Moreover, it was not possible to fully control for all confounding variables (e.g., vaccination status, differences in therapeutic protocols, unreported clinical conditions), which may have influenced the outcomes.

Another limitation is the lack of a long-term follow-up: the analysis focused on 30-day mortality and in-hospital outcomes, without considering survival beyond this period or the functional recovery of patients.

The biggest limitations were as follows: baseline imbalances, absence of adjusted/propensity score-matched studies, limited subgroups, implausibly tight confidence intervals for sample size, and simple fracture classifications.

## Conclusions

COVID-19 appears to have negatively influenced outcomes in elderly patients with hip fractures, with several studies suggesting a possible association with increased short-term mortality. This situation underscores the potential importance of a multidisciplinary approach to what has been described as a "pandemic within a pandemic". Management strategies could benefit from incorporating infection control measures, standardized hospitalization protocols, timely surgical intervention, and structured rehabilitation follow-up. Such approaches may help reduce mortality and adverse events in this particularly vulnerable population as the pandemic continues to evolve.
